# Biglycan Deletion Alters Adiponectin Expression in Murine Adipose Tissue and 3T3-L1 Adipocytes

**DOI:** 10.1371/journal.pone.0050554

**Published:** 2012-11-26

**Authors:** Meliza G. Ward, Kolapo M. Ajuwon

**Affiliations:** Department of Animal Sciences, Purdue University, West Lafayette, Indiana, United States of America; Tohoku University, Japan

## Abstract

Obesity promotes increased secretion of a number of inflammatory factors from adipose tissue. These factors include cytokines and very lately, extracellular matrix components (ECM). Biglycan, a small leucine rich proteoglycan ECM protein, is up-regulated in obesity and has recently been recognized as a pro-inflammatory molecule. However, it is unknown whether biglycan contributes to adipose tissue dysfunction. In the present study, we characterized biglycan expression in various adipose depots in wild-type mice fed a low fat diet (LFD) or obesity-inducing high fat diet (HFD). High fat feeding induced biglycan mRNA expression in multiple adipose depots. Adiponectin is an adipokine with anti-inflammatory and insulin sensitizing effects. Due to the importance of adiponectin, we examined the effect of biglycan on adiponectin expression. Comparison of adiponectin expression in biglycan knockout (bgn^−/0^) and wild-type (bgn^+/0^) reveals higher adiponectin mRNA and protein in epididymal white adipose tissue in bgn^−/0^ mice, as well higher serum concentration of adiponectin, and lower serum insulin concentration. On the contrary, knockdown of biglycan in 3T3-L1 adipocytes led to decreased expression and secretion of adiponectin. Furthermore, treatment of 3T3-L1 adipocytes with conditioned medium from biglycan treated macrophages resulted in an increase in adiponectin mRNA expression. These data suggest a link between biglycan and adiponectin expression. However, the difference in the pattern of regulation between *in vivo* and *in vitro* settings reveals the complexity of this relationship.

## Introduction

Biglycan is a class I member of the small leucine rich proteoglycan (SLRP) family [1] and a component of the extracellular matrix (ECM). Biglycan is associated with bone formation, collagen interaction, and TGF-β signaling [2]–[4]. Biglycan is also able to signal through toll-like receptors in macrophages, leading to secretion of TNFα mature IL-1β and highlighting a pro-inflammatory role for biglycan [5], [6]. Multiple studies show that biglycan is present in the adipose tissue and adipocytes [7]–[10]. Biglycan is expressed in mouse adipose tissue [7], [8], and proteomic and genomic profiling studies show that biglycan gene expression is modulated during adipogenesis, with evidence for increased expression in human mesenchymal stem cells [9] and 3T3-L1 [10] adipocytes. However, proteomic analysis indicates reduced biglycan protein level during adipocyte differentiation [11]. At present it is still unknown whether biglycan contributes to adipose tissue dysfunction in obesity.

Extracellular matrix remodeling is a necessary component for adipose tissue expansion [12]. However, increased extracellular matrix deposition may be a cause of adipose tissue dysfunction during obesity [13]. One way that an extracellular matrix protein could impact adipose tissue function is by affecting the production of adipokines. Adiponectin is an adipokine that is anti-diabetic and promotes fatty acid oxidation. Unlike other adipokines, adiponectin decreases with obesity [14]–[17]. Due to the importance of adiponectin as an adipokine, we wanted to examine the effect of biglycan on adiponectin production. The purpose of this study was first to characterize biglycan expression in various adipose depots in mice fed either low or high fat diet. Next, we examined a possible link between biglycan and adiponectin in different systems. We determined adipose tissue and serum adiponectin levels in biglycan knockout mice and in 3T3-L1 adipocytes with siRNA suppressed biglycan expression. We further examined the effect of biglycan on crosstalk between macrophages and adipocytes using macrophage conditioned medium from biglycan treated RAW 264.7 macrophages. Results of these studies show that adiponectin expression is higher in biglycan knockout mice compared to wild type mice. However, the *in vitro* studies indicate that biglycan may promote adiponectin production. Knockdown of biglycan in 3T3-L1 adipocytes resulted in reduced adiponectin expression, and addition of conditioned medium from biglycan treated macrophages induced adiponectin expression in 3T3-L1 adipocytes. These results show the complexity of the relationship between biglycan and adiponectin expression.

## Materials and Methods

### Animal Use

All animal care and use protocols in this study was approved by the Purdue Animal Care and Use Committee (PACUC). Animals were held under controlled environment at the Purdue small animal housing facility and all efforts were made to minimize discomfort. Heterozygous biglycan females on a C57BL/6J background were obtained from the Mutant Mouse Regional Resource Centers (Columbia, MO, USA) and were crossed onto wild-type C57BL/6J male mice. The progeny were backcrossed to yield wild-type (bgn^+/0^) and knockout (bgn^−/0^) male mice. The biglycan gene is located on the X chromosome; hence, male wild-type mice are bgn^+/0^ and male biglycan null mice are bgn^−/0^. This nomenclature is consistent with the original report characterizing these mice, as well as subsequent reports [18]–[20]. Mice were housed 2–3 to a cage. Animals were genotyped as previously described [21]. At eight weeks of age, bgn^+/0^ and bgn^−/0^ mice were fed either a low fat diet (LFD, 10% kcal fat, #D12450B, Research Diets, New Brunswick, NJ USA) or a high fat diet (HFD, 60% kcal fat, #D12492, Research Diets, New Brunswick, NJ USA) *ad libitum* for 10 weeks (n = 9–12 per treatment group) after which they were euthanized by CO_2_ asphyxiation for blood and tissue collection.

### BMI Calculations

Mice were weighed and measured from the tip of the nose to the start of the tail prior to sacrifice. Body mass index (BMI) measurements were calculated using the following equation for mice: BMI = g/cm^2^ [22], [23].

### Fasting Glucose and Insulin Measurements

Nine weeks into the diet, mice were fasted for 6 hours and fasting blood was collected. Fasting glucose was measured using a Freestyle ™ glucometer system (Abbott, Illinois, USA). Fasting blood was analyzed for serum insulin levels using an insulin enzyme-linked immunosorbent assay (ELISA) (Crystal Chem, Illinois, USA). The following cited in other mouse studies calculation was used to determine the homeostasis model of assessment of insulin resistance (HOMA-IR) index: [fasting plasma level insulin (mU/l) x fasting glucose (mmol/l)]/22.5 [24], [25].

### Primary Cell Collection

Wild-type male C57BL6/J mice, aged 4–5 months, were fed a high fat diet *ad libitum* for 2 weeks (average weight±SE  =  44.56±1.67 g). Mice were fasted for 3 hours and then sacrificed. Epididymal adipose tissue from 4 mice were pooled into buffered saline (0.15 M NaCl, 10 mM HEPES, pH 7.4) for each replicate. The adipose tissue was then minced, rinsed with saline, then transferred to conical tubes containing collagenase type I at a concentration of 100 U/ml in Krebs-Ringer bicarbonate buffer cocktail (10 mM NaHCO_3_, 10 mM HEPES, 5 mM D-glucose, 120 mM NaCl, 4.6 mM KCl, 1.25 mM CaCl_2_, 1.20 mM MgSO_4_, 1.20 mM KH_2_PO_4_, 6% BSA). The collagenase digestion mixture was incubated for 40 min at 37°C with gentle shaking. Cells were separated from large tissue by filtration through a 290 µm screen. The resulting solution was allowed to rest, allowing adipocytes to float to the top. The lower phase was centrifuged to collect stromal vascular cells. Cells were collected into Trizol (Invitrogen, Carlsbad, CA, USA) for RNA extraction.

### 3T3-L1 Cell Culture

3T3-L1 cells (ATCC, Manassas, VA) were differentiated as previously described [26]. Briefly, cells were grown at 5% CO_2_ at 37°C in Dulbecco’s modified eagle media (DMEM) with 10% bovine calf serum supplemented with 1% penicillin-streptomycin mixture. At 2 days post confluence (day 0), cells were differentiated in DMEM with 10% fetal bovine serum containing 1.7 µM insulin, 1 µM dexamethasone, and 0.5 mM isobutylmethylxanthine for 48 hours. Cells were then treated with 10% fetal bovine serum in DMEM containing insulin only for another 48 hours. From this point on, cells were treated with DMEM containing 10% fetal bovine serum.

### siRNA Tranfection of Differentiated 3T3-L1 Cells

To perform the siRNA transfections, 3T3-L1 cells that were 6 days post-differentiation were trypsinized and plated onto siRNA/lipid complexes on 24-well plates at a concentration of 60,000 cells/well in DMEM with 10% fetal bovine serum without antibiotics. siRNA/lipid complexes were formed in wells by incubating 100 µl of Optimem (Invitrogen, Carlsbad, CA, USA) and either 6 pmol of biglycan target siRNA (Santa Cruz Biotechnology, Santa Cruz, CA, USA) or scrambled siRNA (Ambion, Grand Island, NY, USA) and 1µl of Lipofectamine RNAimax (Invitrogen, Carlsbad, CA, USA) at room temperature for 20 min. Cells were incubated at 5% CO_2_ at 37°C for 48 hours, after which the medium was replaced with DMEM containing 10% fetal bovine serum for an additional 48 hours. Medium and cell samples were harvested for analysis.

### Macrophage Conditioned Media Assay

RAW 264.7 cells were grown in media containing DMEM with 10% bovine calf serum supplemented with 1% penicillin-streptomycin mixture until cells reached 50% confluence. RAW 264.7 cells were then incubated in treatment media (DMEM, 0.1% bovine calf serum, 1% penicillin-streptomycin) with or without lipopolysaccharide (100 ng/ml, Sigma-Aldrich, St. Louis, MO, USA) or biglycan (10 µg/ml, bovine origin, Sigma-Aldrich, St. Louis, MO, USA) for 24 hours. Designated treatment groups were treated with LPS (100 ng/ml) in treatment media for 2 hours, after which cells were washed twice in phosphate buffered saline and then treated with or without biglycan (10 µg/ml) in treatment media for 22 hours. After treatments, media was harvested from the RAW 264.7 cells and spun for 3000 g for 5 min. Supernatant from macrophage conditioned media was then added to 3T3-L1 adipocytes 8 days post-differentiation. Adipocytes were incubated in the macrophage conditioned media for 24 hours, after which RNA was harvested for RT-PCR. All conditions were carried out at 5% CO_2_ at 37°C.

### Gene Expression Analysis

Total RNA was extracted from tissues homogenized in Trizol (Invitrogen, Carlsbad, CA, USA) according to the manufacturer’s instructions. RNA concentrations were determined using a Nanodrop reader (Thermo Scientific, Waltham, MA, USA). RNA samples were subjected to gel electrophoresis on a 0.8% agarose gel to check for degradation and genomic DNA contamination. We assessed the expression of select genes through RT-PCR. RNA samples were reverse transcribed using the Reverse Transcription system by Promega (Madison, WI, USA). PCR was performed on the Bio-Rad iCycler. The PCR reaction mix consisted of 0.5 ug of cDNA, 0.075 nmol of each of the forward and reverse primers, and RT^2^ SYBR Green qPCR master mix (SAbiosciences, Frederick, MD, USA); nuclease treated water was added to reach a total reaction volume of 20 µl. Reactions were incubated at 95°C for 5 minutes. Afterwards, the reactions were cycled 40 times using the following protocol: 10 seconds at 95°C, 20 seconds at 55°C, 72°C. The following primers were used for RT-PCR: 18 S (forward-5′ATCCCTGAGAAGTTCCAGCA 3′, reverse 5′-CCTCTTGGTGAGGTCGATGT-3′), biglycan (forward 5′-GACAACCGTATCCGCAAAGT-3′, reverse 5′-GTGGTCCAGGTGAAGTTCGT-3′), adiponectin (forward 5′- GCAGAGATGGCACTCCTGGA-3′, reverse 5′- CAGGGAAGCCTCTTTCTCCT-3′), PPARγ2 (forward 5′-TTGACCCAGAGCATGGTGC-3′, reverse 5′-GAAGTTGGTGGGCCAGAATG-3′) CD68 (forward 5′-GATGTGGAACCCATAACTGGATTCAC-3′, reverse 5′-GGTCCCAGTCTCATTTAGCCACAGTA-3′), FAS (forward 5′GTGAAGAAGTGTCTGGACTGTGTC-3′, reverse 5′-TTTTCGCTCACGTGCACTTTA) TNFα (forward 5′- AGCCCCCAGTCTGTATCCTT-3′, reverse 5′- CTCCCTTTGCAGAACTCAGG-3′), IL-6 (forward 5′-AACGATGATGCACTTGCAGA-3′, reverse 5′- GAGCATTTGGAAATTGGGGTA-3′) and IL-1β (forward 5′-CTAAAGTATGGGCTGGACTG-3′, reverse 5′-GGCTCTCTTTGAACAGAATG-3′). Gene expression was normalized to 18 S using the ΔΔct method.

### Immunohistochemistry

Tissue sections were fixed in 10% neutral buffered formalin and embedded in paraffin wax. 8 µm sections were mounted onto slides and deparaffinized in changes of xylene, 100% ethanol, 95% ethanol and 70% ethanol. Antigen retrieval was performed in sodium citrate buffer (10 mM sodium citrate, 0.05% tween 20, pH 6.0) heated to 95°C for 3 minutes. Sections were blocked in 50 mM Tris buffered saline pH 7.4 (TBS) containing 10% normal donkey serum and 1% BSA at room temperature for 2 hours. After blocking, sections were incubated in primary goat anti-biglycan antibody (Abcam, Cambridge, MA, USA) diluted in TBS containing 1% BSA overnight at 4°C. To control autoflourescence, sections were washed three times in TBS containing 0.025% Triton-X 100 then incubated in a solution of 0.1% sudan black dye diluted in 70% ethanol for 30 minutes at room temperature following primary antibody incubation. Sections were then incubated in Alexa Flour® 488 donkey anti-goat antibody (Invitrogen, Carlsbad, CA, USA) diluted in TBS 1% BSA for 1 hour in the dark at room temperature. Fluorescent images were captured with a Coolsnap HQ CCD camera (Photometrics, Tuscon, AZ, USA) driven by IP Lab software (Scanalytics Inc, Ontario, NY, USA) using a Leica DM6000 microscope (Leica, Buffalo Grove, IL, USA). Background was subtracted from the figures using the rolling ball algorithm (pixel = 50) in Image J (National Institutes of Health, Bethesda, MA, USA). Image and sample processing was performed in tandem. All contrast and brightness adjustments were performed in parallel.

### Serum Adiponectin Measurements

To measure serum adiponectin measurements, we used a Quantikine® adiponectin ELISA kit (R&D Systems, Minnesota, USA) which measures full-length mouse adiponectin.

*LDH assay* Medium from cell culture was measured using the lactate dehydrogenase (LDH) diaphorase kit from Cayman Chemical (Ann Arbor, Michigan, USA).

### Western Blotting

Tissues were homogenized in radio-immunoprecipitation assay buffer and centrifuged at 10,000 g to generate protein samples. Protein concentrations were determined through bicichoninic acid assay (Thermo Scientific, Rockford, IL, USA). Equal protein amounts were resolved on 10% SDS polyacrylamide gels. Proteins were electrophoretically transferred onto nitrocellulose membranes for blotting. Successful transfer was assessed through Ponceau S staining. The following primary antibodies were use: anti-adiponectin (Biovision, Mountain View, CA, USA), anti-β-actin (Cell Signaling Technology, Danvers, MA, USA), anti-biglycan (Abcam, Cambridge, MA, USA), anti-Hsp90 (Cell Signaling Technology, Danvers, MA, USA). Membranes were developed using the Immobilon chemiluminescent HRP substrate kit (Millipore, Billerica, MA, USA).

### Statistics

All statistics were performed using SAS software (SAS institute, Cary, NC, USA).

Data was analyzed using ANOVA through the proc MIXED procedure followed by separation of means by Tukey analysis. If data residuals were non-normal, data was transformed using the Box-Cox procedure. Student’s t-test was used where specified. P-values less than 0.05 were deemed significant.

## Results

### Distribution of Biglycan in Mouse Adipose Tissue

**Figure 1:**
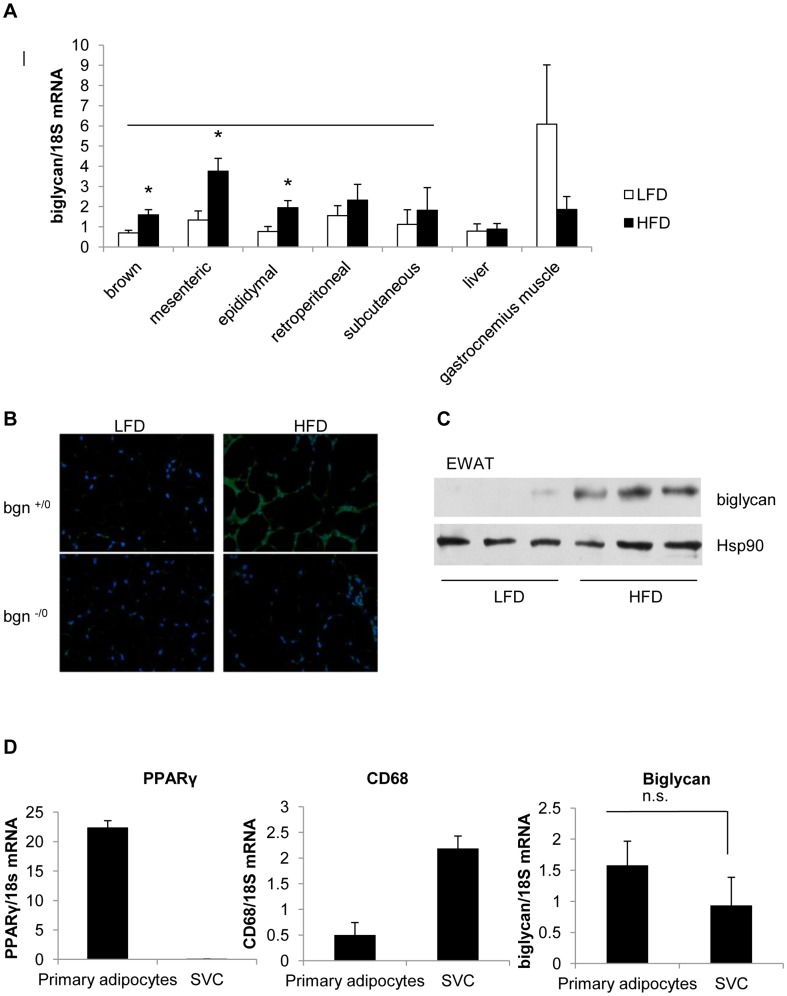
Biglycan expression in adipose tissue. Biglycan mRNA expression in adipose tissue of C57BL6/J wild-type mice fed either LFD or HFD (A). Biglycan expression is elevated in all the adipose depots of HFD mice; however, only mesenteric, brown, and epididymal adipose depots had a significantly higher level. Student’s t-test were performed between LFD and HFD samples (*p<0.05). Sample sizes: brown fat (n = 6), mesenteric adipose (n = 4), epididymal adipose (n = 8), retroperitoneal adipose (n = 8), subcutaneous adipose (n = 6), liver (n = 6), gastrocnemius muscle (n = 4). B) Biglycan staining in EWAT sections from bgn^+/0^ and bgn^−/0^ mice. Magnification 200x, green: biglycan staining, blue: DAPI stain. C) Western blot of biglycan core protein in EWAT. Each lane represents a separate sample. 30 µg of total protein were loaded per lane. D) Expression of biglycan mRNA in primary adipocytes and primary SVC cells from from EWAT of HFD fed wild type mice. PPARγ2 (adipocyte-specific) and CD68 (macrophage-specific) expression levels are used as cell fraction controls. Biglycan expression was not significantly different between cell types (n = 3). For all graphs in figure 1, results are expressed as mean±SE.

Figure 1A shows the gene expression of biglycan in LFD and HFD fed wild-type mice (n = 8–10) in multiple adipose depots, liver and gastrocnemius muscle. Biglycan expression was increased in all adipose depots under HFD. However, the increase in biglycan was significant in only the brown, mesenteric, and epididymal fat pads. No statistical differences were detected in the liver or gastrocnemius muscle. Immunohistochemistry was performed to determine the distribution of biglycan in epididymal white adipose tissue (EWAT) under LFD and HFD bgn^+/0^ mice (fig. 1B). Under LFD, biglycan signal in bgn^+/0^ mice was indistinguishable from background found in bgn^−/0^ mice. However, under HFD, strong biglycan signal appeared around pericellullar areas which are rich in extracellular matrix proteins. Western blot of the core biglycan protein in EWAT confirmed higher amount of biglycan in HFD fed mice (fig. 1C). To determine which cells within the adipose tissue are contributing to biglycan content, we measured biglycan transcript levels in primary adipocytes and stromal vascular cells (SVC) from EWAT of HFD fed wild-type mice. PPARγ and CD68 expression levels were used to confirm cell types (fig. 1D). Transcript levels of biglycan were not significantly different between primary adipocytes and SVC cells.

To determine the adiposity of bgn^+/0^ and bgn^−/0^ mice, first we measured body weight and noted that bgn^+/0^ mice had significantly higher body weights than bgn^−/0^ counterparts (Table 1). However, bgn^−/0^ have altered growth rates after 3 months of age which may impact body weight [18]. When measuring the length of the mice, we found that the bgn^−/0^ mice were shorter in length than the bgn^+/0^ mice. Because the mice were 18 weeks of age at the time of sacrifice, differences in growth rates may have arisen between the genotypes, leading to different body weights between genotypes. To normalize adiposity between genotypes, other measures of adiposity were considered. BMI was not significantly different between the bgn^−/0^ and bgn^+/0^ mice. Additionally, weights of excised adipose depots were expressed as a percentage of total body weight. As expected, high fat diet resulted in increased adipose depot percentages (Table 1). We also observed a significant interaction between genotype and diet in EWAT percentage, where HFD-fed bgn^−/0^ mice had an increased percentage of EWAT when compared to HFD-fed bgn^+/0^ mice, suggesting an increased capacity for EWAT expansion when bgn^−/0^ are HFD-fed. However, there was no genotype effect on the percentages of EWAT, SWAT or RWAT, suggesting similar adiposities between bgn^+/0^ and bgn^−/0^ mice (Table 1).

**Table 1:** Mouse characteristics.

	bgn^−/0^		bgn^+/0^		p-value		
	LFD	HFD	LFD	HFD	genotype	diet	gene × diet
Body weight (g)	28.487±1.061	39.427±1.384	34.046±1.546	45.083±1.930	0.001	<0.001	0.972
Length (cm)	8.555±0.164	9.118±0.109	9.350±0.096	9.700±0.138	<0.001	0.001	0.304
BMI (g/cm^2^)	0.389±0.014	0.477±0.022	0.389±0.015	0.480±0.020	0.827	<0.001	0.838
EWAT (g)	0.681±0.090^a^	1.842±0.138^b^	0.958±0.156^a^	1.495±0.076^b^	0.857	<0.001	0.016
* EWAT %*	2.354±0.254^a^	4.766±0.417^b^	2.852±0.474^a^	3.393±0.225^a^	0.265	<0.001	0.016
SWAT (g)	0.444±0.048	1.574±0.160	0.674±0.94	2.102±0.206	0.012	<0.001	0.283
* SWAT %*	1.545±0.153	4.012±0.390	2.001±0.283	4.614±0.385	0.096	<0.001	0.756
RWAT (g)	0.231±0.029	0.698±0.059	0.359±0.083	0.989±0.091	0.008	<0.001	0.279
* RWAT %*	0.795±0.093	1.787±0.156	0.994±0.190	1.999±0.122	0.144	<0.001	0.872
Gastrocnemius (g)	0.465±0.072	0.536±0.070	0.558±0.071	0.576±0.045	0.607	0.670	0.289
* Gastrocnemius%*	1.619±0.238	1.384±0.203	1.640±0.200	1.306±0.145	0.196	0.005	0.474
Liver (g)	1.186±0.163	1.525±0.204	1.235±0.057	2.116±0.302	0.176	0.013	0.243
* Liver %*	4.132±0.435	3.939±0.593	3.824±0.343	4.724±0.835	0.752	0.538	0.402

Lengths of individual mice were measured from the nose to anus. Organ weights were divided by total body weight to express organs as a percentage of body weight. Results are represented as mean ± SE. EWAT = epididymal white adipose tissue, SWAT = subcutaneous white adipose tissue, RWAT = retroperitoneal white adipose tissue. When a significant diet by genotype interaction was present, means were separated by Tukey analysis and superscript letters are used to indicate significantly different means. P-values less than 0.05 are deemed significant.

**Figure 2:**
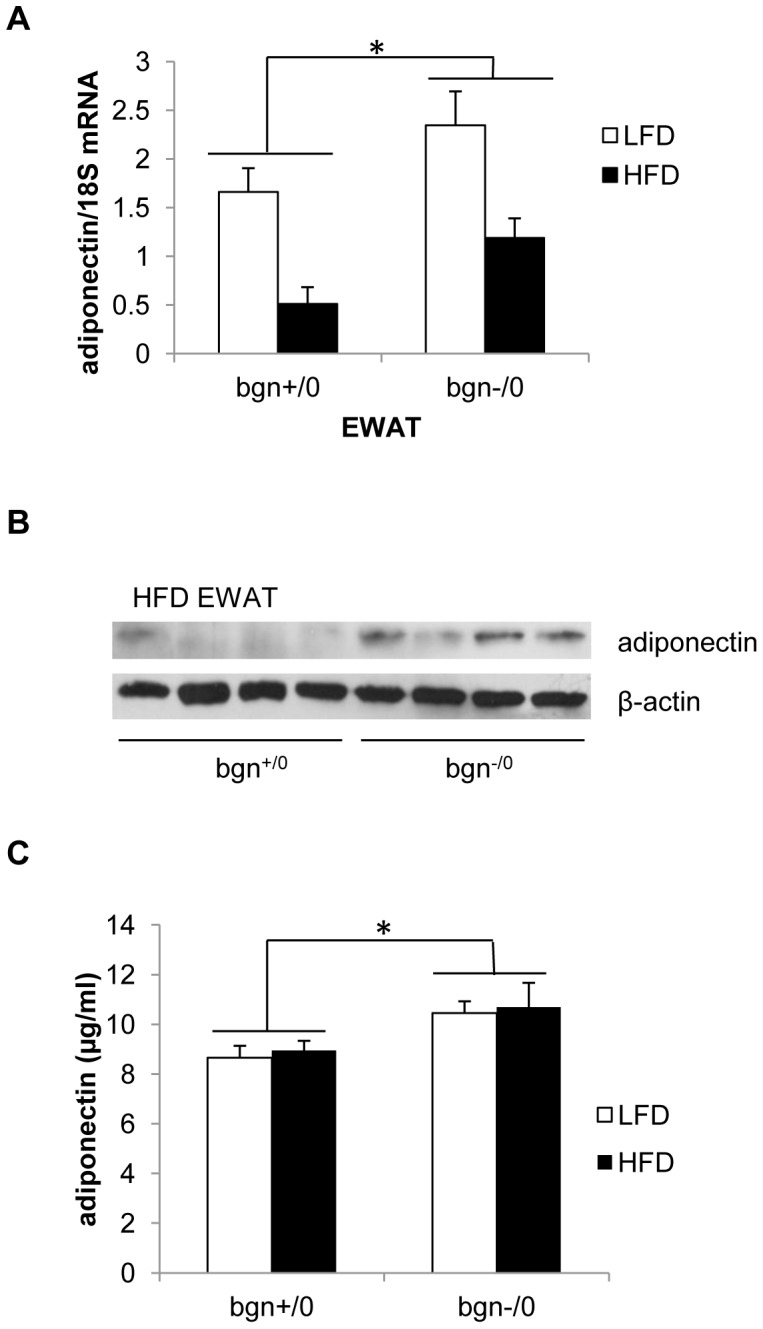
Adiponectin expression in bgn^−/0^ mice. A) Adiponectin mRNA levels in EWAT of bgn^+/0^ and bgn^−/0^ mice on LFD and HFD (n = 9–12). *p<0.05, bgn^−/0^ vs. bgn^+/0^. B) Western blot analysis of protein levels of adiponectin in EWAT in bgn+/0 and bgn−/0 mice fed a high fat diet. Adiponectin levels are normalized to β-actin. Each lane represents one mouse. C) Serum adiponectin levels measured by ELISA (n = 9–12 mice per treatment group), *p<0.05, bgn^−/0^ vs. bgn^+/0^. For all graphs in figure 2, results are expressed as mean±SE.

**Figure 3:**
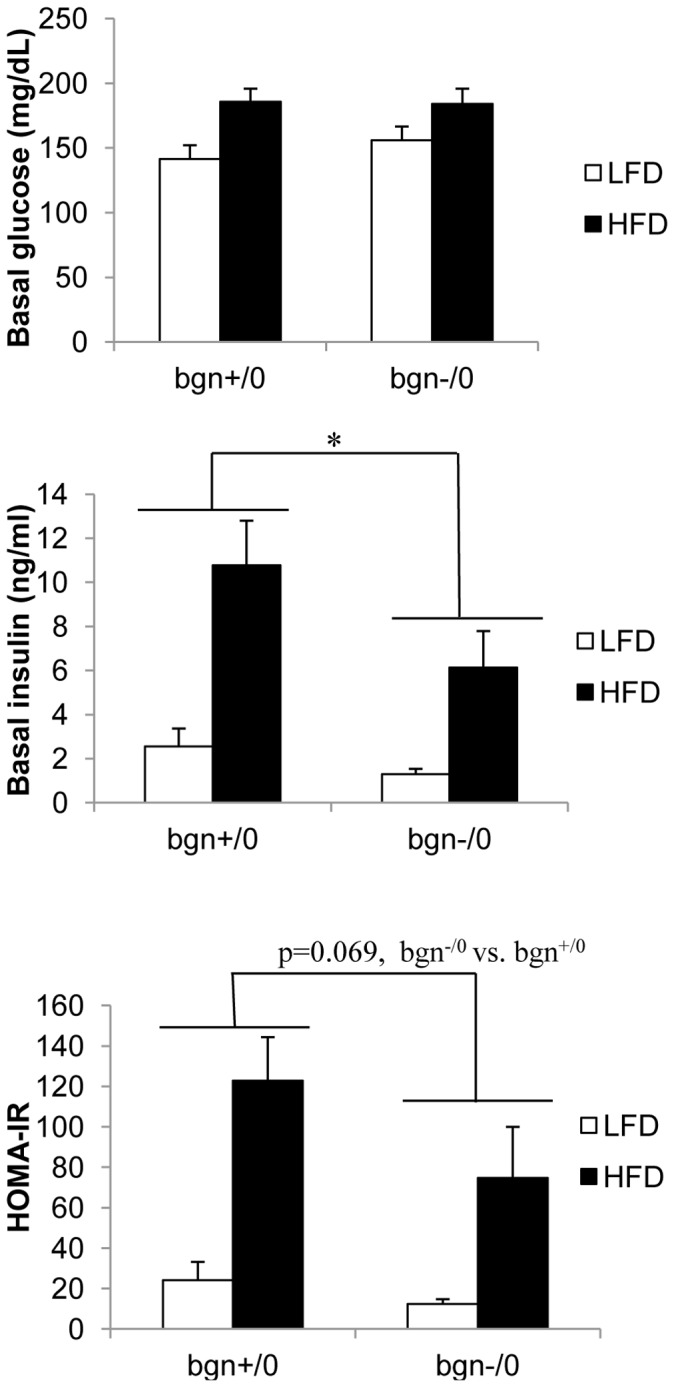
Fasting glucose and insulin. Fasting blood was collected from mice that were fasted for 6 hrs. A) Basal insulin and B) basal glucose of bgn^+/0^ and bgn^−/0^ mice (n = 9–12) *p<0.05 bgn^−/0^ vs. bgn^+/0^. C) HOMA-IR values calculated from basal insulin and basal glucose. Results are expressed as mean±SE.

Adiponectin transcript levels were measured in the EWAT of bgn^+/0^ and bgn^−/0^ mice to determine whether the absence of biglycan affects adiponectin expression. Bgn^−/0^ mice, regardless of diet, exhibited increased adiponectin transcript levels in EWAT when compared to bgn^+/0^ mice (fig. 2A). Next we compared adiponectin protein in EWAT of HFD fed mice (fig. 2B). Bgn^−/0^ mice had higher levels of monomeric biglycan when compared to bgn^+/0^ mice. Circulating levels of adiponectin were also overall increased in the bgn^−/0^ mice when compared to bgn^+/0^ mice (fig. 2C). We did not detect a difference in circulating adiponectin between diets in either the bgn^−/0^ or bgn^+/0^ mice. This result is consistent with other reports that show no decrease in serum adiponectin with long periods of HFD feeding despite changes in adiponectin transcript and protein as well as adiposity [27]–[29]. Because circulating adiponectin is higher in the bgn^−/0^ mice, we hypothesized that bgn^+/0^ and bgn^−/0^ mice may have differing levels of insulin sensitivity. In order to measure insulin sensitivity, we measured fasting insulin, fasting glucose and calculated HOMA-IR. Both fasting insulin and HOMA-IR were significantly affected by diet (p<0.001 for insulin and HOMA-IR) (fig. 3). Fasting insulin was overall significantly decreased in the bgn^−/0^ mice, and HOMA-IR showed a trend (p = 0.069) towards greater insulin sensitivity (lower HOMA-IR values) in bgn^−/0^ mice.

### Transient Knockdown of Biglycan in Mature 3T3-L1 Cells Leads to Decreased Adiponectin Expression

**Figure 4:**
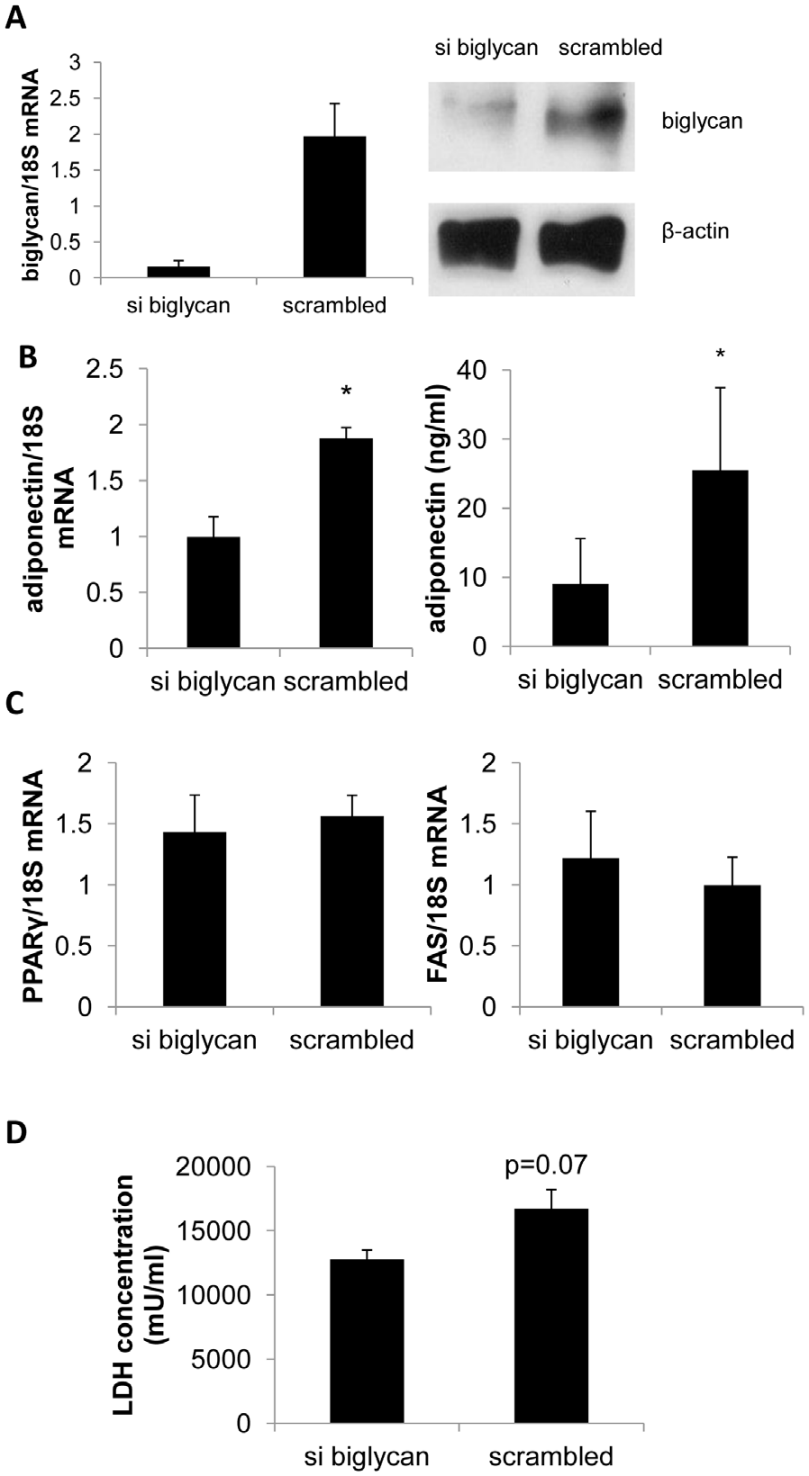
Knockdown of biglycan in 3T3-L1 adipocytes. A) Biglycan expression from 3T3-L1 mature adipocytes treated with siRNA against biglycan (“si biglycan”) or nontargeting siRNA (“scrambled”). Results are from RT-PCR and western blot for core biglycan protein (representative blot). (n = 3 replicates) B) Adiponectin mRNA and secreted measurements from siRNA treated 3T3-L1 adipocytes. (n = 3 replicates) C) PPARγ and FAS expression measured by RT-PCR. (n = 3 replicates) D) Concentration of LDH in the medium of siRNA treated 3T3-L1 adipocytes. For all graphs in figure 4, results are expressed as mean±SE, *p<0.05 target vs. scrambled.

Because adiponectin was increased in the adipose tissue of the bgn^−/0^ mice, we wanted to know if knocking down biglycan expression in 3T3-L1 adipocytes would *directly* lead to an increase in adiponectin expression. Mature 3T3-L1 cells treated with siRNA targeted against biglycan (si resulted in a 91% decrease in biglycan transcript level (fig. 4A). Protein levels of biglycan were also reduced in the biglycan knockdown group (fig. 4A). Both adiponectin transcript and secreted adiponectin were reduced in adipocytes treated with biglycan targeted siRNA (fig. 4B). Adiponectin is primarily secreted by adipocytes and is thus tied to differentiation; hence, reduced adiponectin may result from inadequacies in adipogenesis. Therefore, to clarify if biglycan altered adipocyte differentiation, we measured PPARγ and FAS transcript levels in the treated adipocytes. PPARγ and FAS transcript levels were not significantly different between treatments (fig. 4C). Furthermore, we measured lactate dehydrogenase (LDH) activity to determine if there was difference in cytotoxicity from the biglycan and scrambled siRNA. We instead found a trend towards lower LDH activity in the cells in which biglycan was suppressed (si biglycan, fig.4C). These results indicate that the observed decrease in adiponectin was not due to hampered adipogenesis or cytotoxicity. To determine whether inflammation was affected, we also measured IL-6 transcript level in the siRNA treated 3T3-L1 adipocytes. We did not observe a difference in IL-6 transcript when biglycan was suppressed (data not shown).

### Biglycan Treated Macrophage Conditioned Media Illicit Increases in Adiponectin Expression

**Figure 5:**
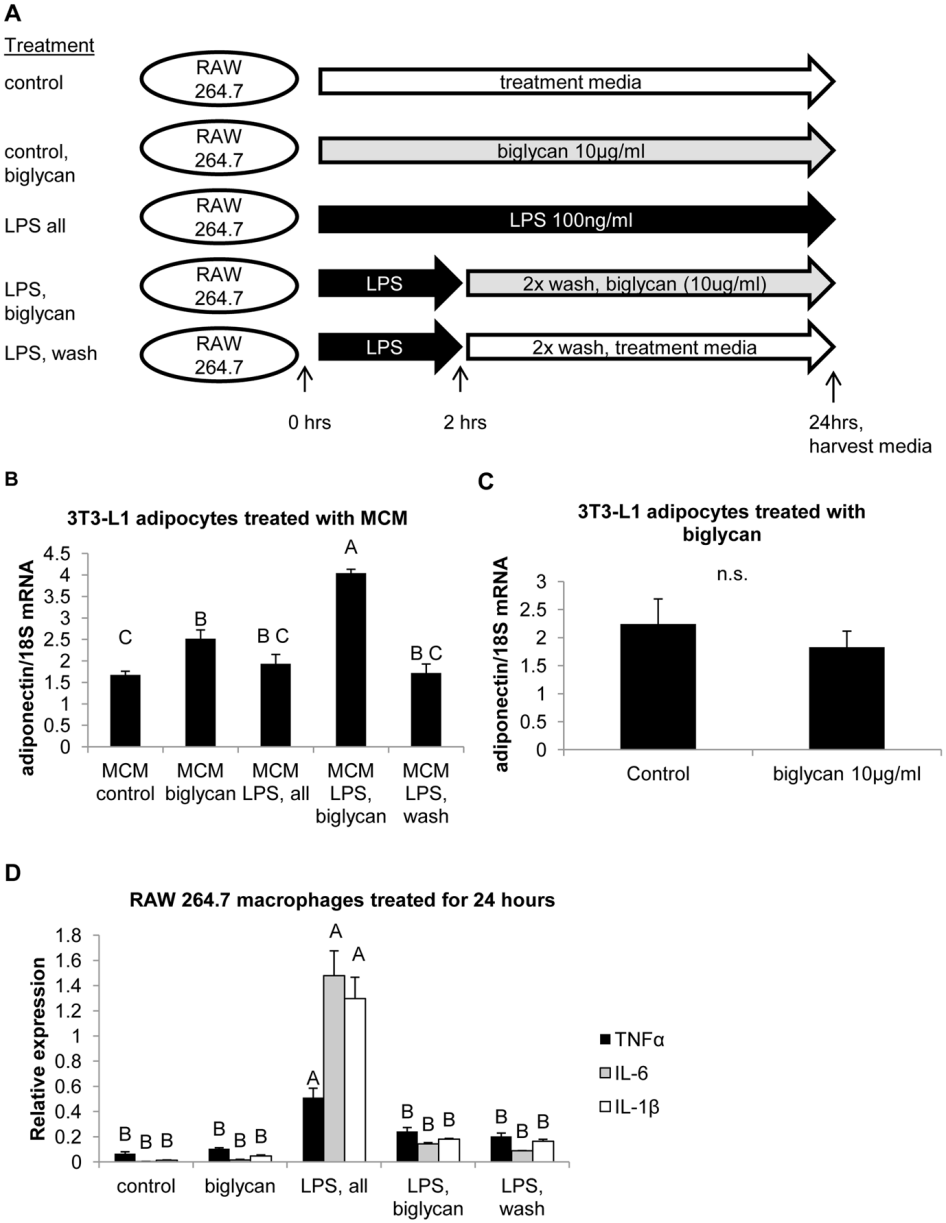
Biglycan treated RAW264.7 macrophages and 3T3-L1 adipocytes treated with macrophage conditioned medium (MCM). A) Diagram of treatment scheme. RAW 264.7 macrophages were treated with combinations of treatment media (control), biglycan, or LPS. Some treatment groups were primed with LPS for two hours. Macrophage conditioned medium (MCM) from the treatments was used to treat 3T3-L1 adipocytes. B) Adiponectin mRNA in 3T3-L1 adipocytes treated with MCM. Eight days after differentiation, 3T3-L1 adipocytes were treated with MCM from RAW 264.7 macrophages for 24 hours. (n = 3 replicates) Statistically different means were determined by Tukey means separation after ANOVA and are denoted by different letters above bars in the graph. C) Adiponectin mRNA from 3T3-L1 adipocytes (8 days post-differentiation) after treatment with biglycan for 24 hours. (n = 3 replicates) n.s.  =  not significant D) mRNA levels of TNFα, IL-6 and IL-1β from macrophages treated with combinations of LPS and biglycan (outlined in figure 5A). All genes measured in figure 5D are expressed relative to 18 S. (n = 3 replicates) Statistically different means were determined by Tukey means separation after ANOVA and are denoted by different letters above bars in the graph. For all graphs in figure 5, results are expressed as mean±SE.

Increased adiposity is associated with increased infiltration of macrophages [30]. Due to the close proximity of macrophages to adipocytes in adipose tissue, we tested whether biglycan mediated crosstalk existed between macrophages and adipocytes. Peritoneal macrophages do not express biglycan without stimulation [19]; similarly, we did not detect biglycan expression in RAW264.7 cells through RT-PCR (data not shown). We treated RAW264.7 macrophages with combinations of lipopolysaccharide (LPS) and biglycan as listed in figure 5A. Because RAW264.7 macrophages are naïve, RAW264.7 cells were first pre-treated with LPS for 2 hours for activation before treatment with biglycan. Activation of macrophages with LPS did not induce biglycan expression (data not shown). When 3T3-L1 differentiated adipocytes were treated with macrophage conditioned media (MCM), MCM from biglycan treated macrophages induced higher adiponectin expression than in 3T3-L1 adipocytes treated with MCM without biglycan (figure 5B). In addition, treatment of 3T3-L1 adipocytes with MCM from RAW264.7 macrophages that had been treated with LPS and biglycan resulted in higher adiponectin mRNA when compared to adipocytes treated with MCM from macrophages that were treated with LPS alone (figure 5B). However, direct treatment of 3T3-L1 adipocytes with biglycan had no effect on adiponectin expression (figure 5C). These data indicate that biglycan can indirectly act through macrophage conditioned medium to influence adiponectin expression, implicating a potential role for biglycan in the crosstalk between macrophages and adipocytes. Next, we measured transcript levels of TNFα, IL-6, and IL-1β in the treated RAW 264.7 cells to determine if the adiponectin response was due to changes in inflammatory cytokines coming from the RAW 264.7 cells. However, as shown in figure 5D, there were no significant changes in TNFα, IL-6, or IL-1β mRNA levels in the RAW 264.7 macrophages due to biglycan treatment.

## Discussion

Extracellular matrix (ECM) components display dynamic expression during obesity. The ECM is remodeled during adipose tissue growth and the expression of the ECM is generally increased in obese individuals [31]–[33]. In this study, we demonstrate that the expression of biglycan is generally increased in adipose tissue of HFD fed obese mice, indicating that biglycan is associated with increased adipose tissue expansion. In addition, we show that both adipocytes and SVC cells contribute to adipose tissue biglycan. An increase in biglycan expression due to HFD was observed in earlier results by Huber, who reported elevated biglycan transcript levels in adipose tissue of high saturated fat fed *db/db* mice [8]. We have previously reported increased biglycan mRNA in the adipose tissue of obese humans [34]. A recently published report demonstrated high expression of biglycan in various adipose depots of *Psammomys obesus* [35]. Furthermore, increased expression of biglycan was associated with impaired glucose tolerance and obesity in *P. obesus,* and we recently reported improved glucose tolerance in biglycan knockout mice on HFD relative to controls [35], implicating a possible role for biglycan in glucose metabolism.

Adiponectin is an anti-diabetic hormone secreted from adipose tissue [15]. Unlike many adipokines, adiponectin expression decreases with increased BMI in humans [36], [37]. Our results show an increase in circulating adiponectin in the bgn^−/0^ mice. We show that while bgn^+/0^ mice have higher body weights, their adiposity and BMI is similar to bgn^−/0^ mice indicating that the observed difference in adiponectin found in the bgn^−/0^ mice is not due to differences in adiposity. Little is known about the interaction between biglycan and adiponectin, although it is reported that biglycan can bind directly to adiponectin [38].

Our results show lower fasting insulin in the circulation of bgn^−/0^ mice and a trend towards increased insulin sensitivity as measured through HOMA-IR. As we have used whole body knockout mice, it is impossible to state at this time that the increase in adiponectin found in the bgn^−/0^ mice is responsible for the observed decrease in fasting insulin. However, the initial finding of a possible improvement in insulin sensitivity in bgn^−/0^ mice opens additional avenues for future research.

While the data from the bgn^−/0^ mice suggest that the absence of biglycan promotes adiponectin expression in a whole body knockout system, *in vitro* results indicate that biglycan absence is associated with a decline in adiponectin expression. Transient knockdown of biglycan led to decreased adiponectin production *in vitro*. Furthermore, medium from RAW 264.7 cells treated with biglycan increased adiponectin expression in 3T3-L1 adipocytes. Biglycan is a pro-inflammatory signal and its presence may induce increases in cytokine expression in RAW264.7 cells. We did not detect a difference in expression of TNFα, IL-6, or IL-1β due to biglycan in RAW264.7 cells that could explain the increase in adiponectin expression found in the 3T3-L1 adipocytes. However, a thorough analysis of secreted factors by the macrophage in response to biglycan is needed to understand whether any pro-inflammatory signals are produced as a mediating factor.

One possible explanation for the difference between the *in vivo* and *in vitro* system may be that the effect of biglycan absence stems from interactions with the extracellular matrix which are absent in a 2-D cell culture. Specifically, biglycan absence may disrupt collagen formation in adipose tissue. Collagen VI null mice have an improved metabolic phenotype which may be attributable to decreased rigidity and fibrosis in adipose tissue [13]. A mechanism may exist whereby biglycan absence inhibits proper collagen VI formation as biglycan is implicated in the organization of collagen VI into hexagonal networks *in vitro* [39].

Although it is unclear at this point the reason for the discordance between the *in vivo* and *in vitro* systems in terms of adiponectin production, it is clear that cellular and tissue level mechanisms are in place to sense the presence of biglycan and regulate adiponectin expression accordingly. Since there are multiple cell types in place in the *in vivo* setting, the lack of biglycan may create a microenvironment that supports increased adiponectin expression or relieve the inhibitory function of negative mechanisms that suppress adiponectin expression. Several studies have linked increased oxidative stress and inflammation to reduced adiponectin expression [40]–[42]. Since activation of PPARγ is associated with increased adiponectin expression [43], inhibition of PPARγ by nuclear factor kappa B (NFκB) during obesity [44] could be a potential link between obesity and reduced adiponectin expression. We have provided evidence that biglycan knockout mice have reduced adipose tissue inflammation indicated by lower expression of inflammatory markers such as IL-6, TNFα and CD68 [7], [45]. Thus the lack of biglycan in the knockout mice will prevent the inhibitory effect of inflammation on adiponectin expression, hence the higher adiponectin expression in the bgn^−/0^ mice. On the other hand, suppression of biglycan *in vitro* in 3T3-L1 cells may send a yet unknown signal into the cell that suppresses adiponectin expression. Since biglycan and adiponectin interact leading to sequestration of adiponectin [39], the lack of biglycan might indicate that less adiponectin is needed for the same level of available adiponectin for bioactivity. Additionally, culture of cells *in vitro* on plastic does not perfectly replicate the *in vivo* conditions of adipocytes in adipose tissue due to the absence of other cellular and non-cellular tissue components. Furthermore, knock down of biglycan in 3T3-L1 adipocytes did not lead to an alteration in the inflammatory state of the cells, marking another major difference between biglycan absence in the *in vivo* and *in vitro* models. Instead, the expected disruption of collagen matrix formation in the 2-D culture condition in the absence of biglycan on plastic surface [39] could affect the integrity of the extracellular matrix, and perhaps extracellular matrix characteristics that may be necessary for adiponectin expression. The induction of adiponectin in adipocytes treated with MCM from biglycan and biglycan and LPS treated macrophages may suggest that these treatments lead to production of yet unidentified factors that induce adipocytes to increase adiponectin expression. What remains constant through both the *in vivo* and *in vitro* results is that the absence of biglycan can impact adiponectin expression, implicating a mechanism where adipocytes can sense biglycan abundance to regulate adiponectin production.

In summary, our findings show an increase in biglycan expression in adipose tissue during obesity. We also observed a modest increase in adiponectin in bgn^−/0^ mice; however, transient knockdown of biglycan in 3T3-L1 cells resulted in decreased adiponectin expression. These studies indicate a complex mechanism by which adipocytes are able to sense biglycan presence in both *in vivo* an *in vitro* settings to regulate adiponectin expression. Further work will be needed to clarify the true nature of this relationship.
